# Enhancing Wheat Disease Diagnosis in a Greenhouse Using Image Deep Features and Parallel Feature Fusion

**DOI:** 10.3389/fpls.2022.834447

**Published:** 2022-03-10

**Authors:** Zhao Zhang, Paulo Flores, Andrew Friskop, Zhaohui Liu, C. Igathinathane, X. Han, H. J. Kim, N. Jahan, J. Mathew, S. Shreya

**Affiliations:** ^1^Key Laboratory of Modern Precision Agriculture System Integration Research, Ministry of Education, China Agricultural University, Beijing, China; ^2^Key Lab of Agricultural Information Acquisition Technology, Ministry of Agriculture and Rural Affairs, China Agricultural University, Beijing, China; ^3^Department of Agricultural and Biosystems Engineering, North Dakota State University, Fargo, ND, United States; ^4^Department of Plant Sciences, North Dakota State University, Fargo, ND, United States; ^5^Department of Biosystems Engineering, College of Agriculture and Life Sciences, Kangwon National University, Chuncheon, South Korea; ^6^Interdisciplinary Program in Smart Agriculture, College of Agriculture and Life Sciences, Kangwon National University, Chuncheon, South Korea; ^7^Department of Biosystems and Biomaterials Engineering, College of Agriculture and Life Sciences, Seoul National University, Seoul, South Korea; ^8^Department of Electrical and Computer Engineering, North Dakota State University, Fargo, ND, United States

**Keywords:** wheat disease, plant pathology, deep features, handcrafted features, data fusion

## Abstract

Since the assessment of wheat diseases (e.g., leaf rust and tan spot) *via* visual observation is subjective and inefficient, this study focused on developing an automatic, objective, and efficient diagnosis approach. For each plant, color, and color-infrared (CIR) images were collected in a paired mode. An automatic approach based on the image processing technique was developed to crop the paired images to have the same region, after which a developed semiautomatic webtool was used to expedite the dataset creation. The webtool generated the dataset from either image and automatically built the corresponding dataset from the other image. Each image was manually categorized into one of the three groups: control (disease-free), disease light, and disease severity. After the image segmentation, handcrafted features (HFs) were extracted from each format of images, and disease diagnosis results demonstrated that the parallel feature fusion had higher accuracy over features from either type of image. Performance of deep features (DFs) extracted through different deep learning (DL) models (e.g., AlexNet, VGG16, ResNet101, GoogLeNet, and Xception) on wheat disease detection was compared, and those extracted by ResNet101 resulted in the highest accuracy, perhaps because deep layers extracted finer features. In addition, parallel deep feature fusion generated a higher accuracy over DFs from a single-source image. DFs outperformed HFs in wheat disease detection, and the DFs coupled with parallel feature fusion resulted in diagnosis accuracies of 75, 84, and 71% for leaf rust, tan spot, and leaf rust + tan spot, respectively. The methodology developed directly for greenhouse applications, to be used by plant pathologists, breeders, and other users, can be extended to field applications with future tests on field data and model fine-tuning.

## Highlights

– Parallel feature fusion of different types of images improved the accuracy of wheat disease diagnosis.
– Deep features outperformed handcrafted features in wheat disease detection.
– Deep features extracted by deep-layered models produced higher accuracy.
– A free semiautomatic webtool for expedited paired dataset creation was developed and made available.

## Introduction

Wheat (*Triticum aestivum* L.) is one of the world’s most productive and important crops, which plays a crucial role in food security (Curtis and Halford, 2014; Shewry and Hey, 2015). Currently, wheat production faces a number of challenges, among which diseases are ranked among the top (Bolton et al., 2008). In addition to reducing yield, wheat diseases could lower the grain quality or even result in grain contamination due to toxins produced by pathogens (Lu et al., 2017; Qiu et al., 2019). Leaf rust and tan spot are common diseases that affect wheat production in the United States and worldwide, which can cause wheat yield losses of 10–40% (De Wolf, 2008; Sharma et al., 2016).

The two main approaches to manage wheat diseases are breeding disease-resistant varieties and through chemical applications (Kolmer, 1996; Ransom and McMullen, 2008; Figlan et al., 2020). For both approaches, researchers conduct extensive greenhouse work before transferring the most promising materials or treatments to the field for further evaluation. Hence, it is critical for researchers to obtain accurate information on the disease conditions in the greenhouse (Abdulridha et al., 2020). The current approach of wheat disease diagnosis relies on visual observations by well-trained graders. This approach potentially suffers from subjectivity (grader bias), inefficiency (slow speed of observation), inter-grader variation (inconsistent results among different graders), and fatigue (tiresome operation) (Lehmann et al., 2015). Therefore, an automated, efficient, and objective approach to accurately and quickly diagnose wheat diseases is needed (Luvisi et al., 2016).

Leaf rust is characterized by the presence of rust-colored pustules erupting at the crop leaves (Salgado et al., 2016). Tan spot symptoms are oval or diamond-shaped to elongated irregular spots on the leaf, and these spots enlarge and turn tan with a yellow border and a small dark brown spot near the center (McMullen and Adhikari, 2009). Many studies have taken advantage of these visible symptoms and used color [red, green, blue (RGB)] images, coupled with different classifiers, for disease detection (Johannes et al., 2017; Lu et al., 2017; Saleem et al., 2019; Su et al., 2020). Color images are the dominant type of images used for crop disease detection because of their low cost and easiness to acquire and handle (Gaikwad and Musande, 2017; Barbedo, 2018; Qiu et al., 2019; Wiesner-Hanks et al., 2019). In addition to color images, color-infrared (CIR) images have been extensively used in crop disease detection (Lehmann et al., 2015). Different from the color images consisting of RGB, CIR images include three bands, namely, near-infrared (NIR), red, and green bands. The CIR images take advantage of the fact that disease lesions (chlorotic or necrotic) cause biochemical changes on the plant tissue, which can significantly affect the energy reflection on NIR of the electromagnetic spectrum (Roberts et al., 1993; Franke and Menz, 2007; Azadbakht et al., 2019). Healthy plants on the CIR images usually display high reflectance on the NIR band and low reflectance on the red band, while an opposite band reflectance pattern is observed on the unhealthy plants (Carlson and Ripley, 1997). Based on this principle, vegetation indices can be calculated from CIR images. Among them, the Normalized Difference Vegetation Index (NDVI) has been extensively used for monitoring the crop health condition (Bravo et al., 2003; Franke and Menz, 2007; Su et al., 2018; Yang, 2020). CIR images were utilized for wheat and cotton disease detection, with practical application of the results (Bajwa and Tian, 2001, 2002; Moshou et al., 2004; Yang, 2020). Although both RGB and CIR images have been extensively used for crop disease detection, few studies were conducted to compare their performance on wheat disease detection and further improve the methodology and detection accuracy.

After collecting color or CIR images, handcrafted features (HFs), which are extracted from images using algorithms to represent the physical characteristics of the plants, would serve as the basis for classification purposes (Zhang et al., 2016, 2020b; Jahan et al., 2020). Numerous studies associated with crop disease diagnosis have been carried out based on the HFs, including vegetation indices (Ashourloo et al., 2014; Chen et al., 2018), texture (Wood et al., 2012; Sun et al., 2019; Wan et al., 2020), and color (Patil and Kumar, 2011; Gaikwad and Musande, 2017). The HFs-based classification requires domain knowledge on feature selection, as the classification accuracy mainly determines whether the selected features have a good representation of the diseases (Zhang et al., 2020a). One approach to get rid of the domain knowledge required by the HFs-based classification is through deep learning (DL).

During the last decade, DL has experienced significant progress regarding image classification, with the convolutional neural networks (CNNs) having been the core (Zhang et al., 2020b). The CNNs enabled the implementation of algorithms for automatic feature extraction, which does not require domain knowledge. Very recently, deep features (DFs; features automatically extracted by CNNs) have been used in crop disease detection, and the literature in this field is limited. Lu et al. (2017) extracted DFs and applied them to discriminate wheat diseases, such as leaf blotch, smut, stripe rust, and black chaff, but the algorithms’ performance on disease severity diagnosis was not reported. In addition, DFs have been used for the detection of apple scab disease (Khan et al., 2018) and rice leaf disease (Sethy et al., 2020). However, few studies have reported the application of DFs to differentiate and assess the severity of wheat leaf rust and tan spot diseases. Furthermore, the diagnosis accuracies based on selected features (HFs and DFs), which influence classification performance, were unavailable.

Features (usually from a single-source image) were extracted and then fed into classifiers for classification (Yang et al., 2003; Sethy et al., 2020). Features extracted from color images were used to detect tomato leaf diseases (Patil and Kumar, 2011) and corn diseases (Wiesner-Hanks et al., 2019), while CIR images were used for assessing cotton rot disease (Yang, 2020). Since a certain type of image might only provide partial information to aid plant diseases classification, researchers (Bulanon et al., 2009; Castanedo, 2013) had been experimenting with data fusion techniques by combining features from different types of images to improve the model accuracy. Color image blended with NIR image detected the freshness level of food products and demonstrated an improved classification accuracy over either single-source image (Huang et al., 2016). Integration of color and thermal images improved the field orange detection accuracy (Bulanon et al., 2009). In the color images, oranges were not well differentiated from leaves because of similarities between them. However, they had different temperatures, which were obtained *via* thermal images. Thus, the fusion of the features from color and thermal images led to a higher accuracy. Even though the use of data fusion techniques has resulted in higher classification accuracy, few studies have fused color and CIR images information for wheat disease diagnosis.

With an overall goal of developing and implementing an automated solution to assess greenhouse wheat diseases (e.g., leaf rust, tan spot, and leaf rust + tan spot), this study proposes an innovative methodology of using deep features and their parallel fusion from color and CIR images. Specific objectives of this study were: (1) to compare the performance of features from color and CIR images, and their parallel fusion in wheat disease diagnosis; (2) to compare the accuracies of DFs extracted from different DL models on wheat disease diagnosis and select the one generating the highest accuracy; and (3) to compare the accuracies of HFs and DFs in wheat disease detection.

## Materials and Methods

**FIGURE 1:**
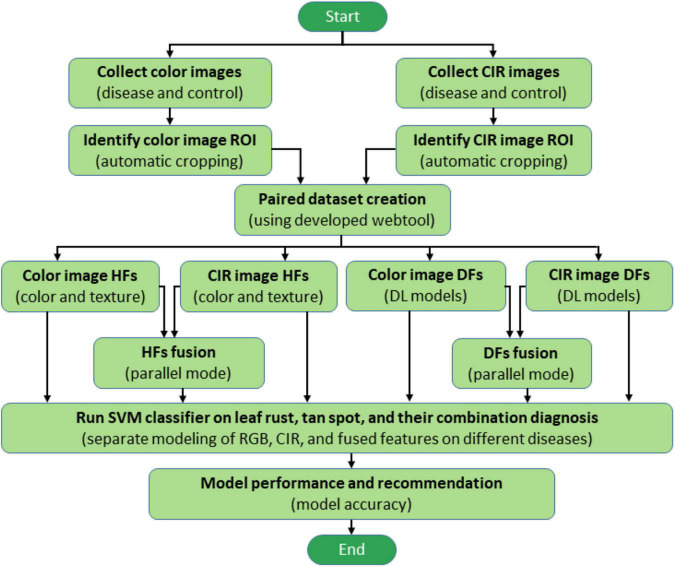
Overall process flowchart of wheat disease diagnosis. ROI, CIR, DL, HFs, DFs, and SVM represent the region of interest, color-infrared, deep learning, handcrafted features, deep features, and support vector machine, respectively.

The various process steps followed in this study to improve wheat disease diagnosis accuracy using feature fusion and DFs are illustrated in Figure 1. After collecting color and CIR images for the same plants in a paired mode, the region of interest (ROI) was automatically determined using image processing techniques. We developed a webtool to expedite the dataset generation—while manually cropping either type of images (color or CIR), the corresponding image of the other type would be generated automatically (paired dataset). After generating the dataset, features (HFs and DFs) extracted from single-source images (color or CIR) and their fusion were fed into a support vector machine (SVM) for accuracy comparison. Finally, the methodology yielding the highest accuracy would be recommended for future application. The following sections describe the processes in detail.

### Image Acquisition

The experiment was conducted at the North Dakota State University, Agricultural Experiment Station Research Greenhouse Complex (Fargo, ND, United States). Since the greenhouse is enclosed with a transparent roof and windows, the crop growing light conditions can be considered as semi-natural illumination. Two wheat varieties (*Prosper* for leaf rust disease and *Jerry* for tan spot disease) were planted in pots (Deepot D40: 6.4 × 25.4 cm; Stuewe and Sons, Inc., Tangent, Oregon). For the two disease groups, crops were properly inoculated and then kept in the incubation chamber for 24 h for expedited development of the diseases. The control group was kept in another incubation chamber with the same conditions and time as the disease group. After inoculation, the two diseases required different amounts of time to display symptoms—about 10 days for leaf rust and 6 days for a tan spot. Immediately after the initial observation of disease symptoms, the data collection started and continued for the next 12 consecutive days, with images being collected between 10:00 a.m. and 12:00 p.m.

**FIGURE 2:**
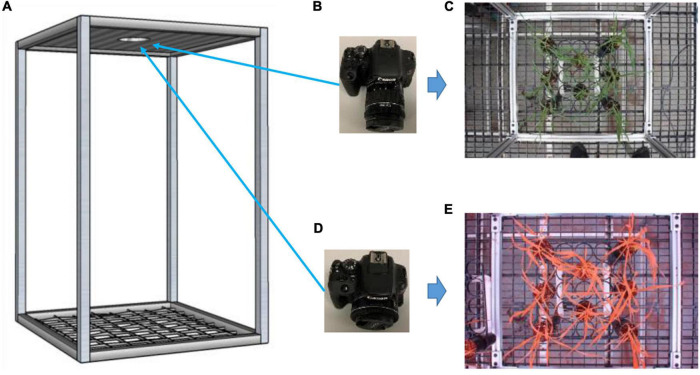
Experimental setup for image collection. A fabricated frame (**A**; 60 × 60 × 90 cm) to hold cameras on the top at the same locations and hold rack of pots at the bottom; a color camera **(B)** with a sample collected image **(C)**; and a color-infrared camera **(D)** with a sample collected image **(E)**.

Two off-the-shelf cameras were used for data collection—a Canon EOS Rebel T7i camera (Ota City, Tokyo, Japan) for color images (6,000 × 4,000 pixel resolution) and a multi-band camera (LDP LLC, Carlstadt, NJ, United States) for CIR images (5,184 × 3,456 pixel resolution) (Figure 2). A frame (60 × 60 × 90 cm) built to facilitate image collection was used as a reference for image collection for both cameras—the diameter of the hole at the top sheet of the frame was a little wider than the diameter of the camera lens, allowing them to go through to capture the images. A rack that could hold 8 pots was placed at the center of the frame bottom for the collection of both color and CIR images, after which the rack was replaced. That process was repeated until all plants were imaged. There were 10 racks for each variety of plants, for a total of 160 pots of plants (8 pots × 10 racks × 2 varieties).

### Color and Color-Infrared Image Datasets Creation

#### Automatic Raw Image Cropping

**FIGURE 3:**
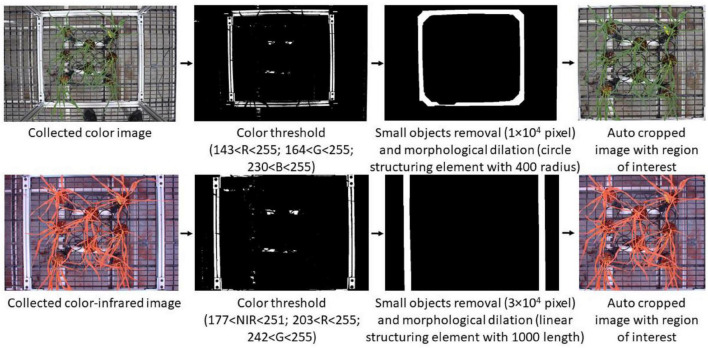
Automatic procedures of using image process techniques to generate the corresponding two types of images with the same view. NIR, near-infrared; R, red; G, green; B, blue.

After collecting color and CIR images, the next step was to prepare the paired datasets (the same portion of plants shown in both color and CIR images). A critical requirement during the paired dataset preparation was to have the color image to be corresponding to the CIR image. Since the two cameras had different field of views and resolutions, as well as the image collection positions were not exactly the same for the two cameras, the views of the two images were different, as shown in Figures 2C,E. It is thus necessary to keep the views of the two images the same for further paired dataset generation by proper cropping. Previous research used a manual approach for image cropping (Bulanon et al., 2009), which is inefficient and inaccurate. In this study, an automatic raw image cropping approach was developed and applied, which used the aluminum square base of the experimental frame as a reference. The frame was first detected using color thresholding, and then, the mask was generated (after noise removal) for each type of image. Only the image section within the square base was kept, and detailed procedures and parameters for image processing are shown in Figure 3.

#### Webtool for Paired Image Dataset Creation

**FIGURE 4:**
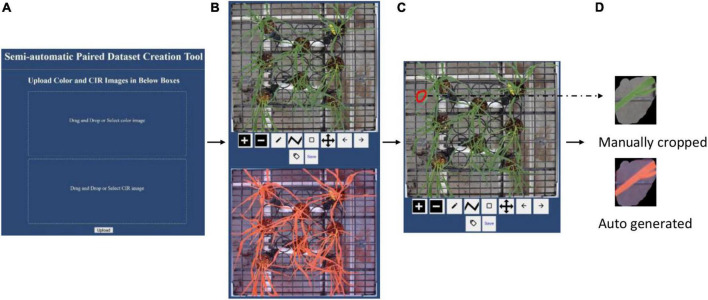
Introduction to the webtool developed to expedite the paired dataset generation accurately. **(A)** Graphical user interface, **(B)** uploaded a pair of images with different formats (color and color-infrared), **(C)** manual image cropping on color image (red irregular shape), and **(D)** sample of a paired dataset.

After the paired images were automatically cropped, they covered the same view. While creating the paired dataset, it required certain plant regions to be present in the paired color and CIR images. Manually processing the images to create the paired dataset presents many issues: (i) manually cropping both the color image and the CIR image can be a laborious process; (ii) users’ manual switching between the two images is inefficient; and (iii) manual cropping method is inaccurate since it is very difficult to replicate the same ROI onto the corresponding image. To address these issues, we developed a webtool that can expedite the workflow and improve the process accuracy. The graphical user interface (GUI) of the developed webtool is shown in Figure 4A and can be accessed *via* this webpage.^1^ Following the GUI instructions, users needed to upload a pair of images (auto-cropped color and CIR image; Figure 3). The webtool would then automatically resize the two images to make their dimensions similar (image size; Figure 4B). Then, users can draw the ROI (any closed polygonal or irregular shape) at the top image (red irregular shape in Figure 4C), after which the corresponding image of the other type would be generated and saved (a sample pair shown in Figure 4D). This free webtool can be accessed by users for a similar image processing workflow.

#### Visual Disease Classification

After the image dataset for each type of disease was generated, each pair of the image was visually classified into one of the three following classes: control (disease-free), disease light (light infection), and disease severe (severe infection). The standards used for classifying leaf rust disease grade are shown in Supplementary Appendix I: If no disease symbol was shown, it was classified as control; if the rust severity level was below 10 of the *modified Cobb Scale* B, it was classified as disease light; otherwise, it was classified as disease severe (Peterson et al., 1948; Gebremariam et al., 2016). Samples of visually graded leaf rust diseases with different severity levels are shown in Supplementary Appendix I. For the tan spot visual grading, the following protocols were followed: If no disease symbol was shown, it was classified as control; if the disease area (discolored portion) was less than 30% of the total leaf area, it was classified as disease light; otherwise, it was classified as disease severe. Samples of visually graded tan spot diseases with different severity levels are shown in Supplementary Appendix II. Since visual disease classification requires domain knowledge, in this study, three individual graders were trained by professional plant pathologists and then voted for the classification of each image. For each image, the grade with more than two votes was assigned as the final grade. There were no cases that the three graders assigned three different grades for the same image.

#### Segmentation of Color and Color-Infrared Images

**FIGURE 5:**
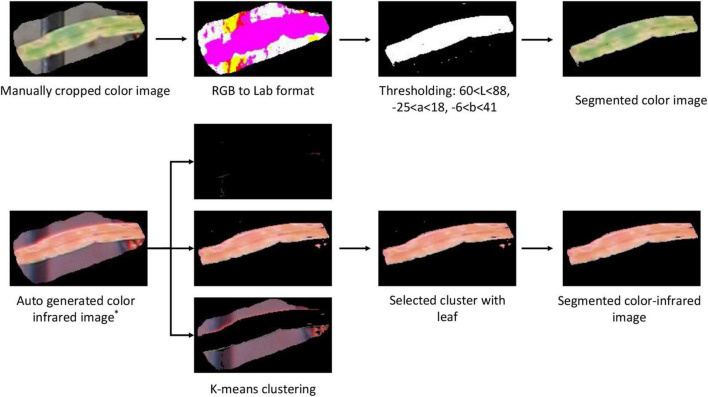
Image segmentation procedures of the color and color-infrared paired images. * refers to the auto-generated image from the webtool (Figure 4).

After creating and grading the datasets, the segmentation of the plant from the noisy background, including fertilizer, peat, plastic grid, and aluminum frame, was performed. Color images were first converted to Lab (*L* for lightness and *a* and *b* for the color dimensions) format, and then, proper thresholding was applied to generate a binary image. After removing small area noises, a binary mask was generated, which was applied to the original image to obtain the segmented color image (Figure 5).

Since the paired color and CIR images are of the same size, the easiest approach to segment the CIR image was to directly apply the binary mask generated during the color image segmentation to the corresponding CIR image. However, considering that most studies used the raw CIR images for disease detection (Bajwa and Tian, 2002; Yang, 2020) and few studies reported CIR image segmentation methods, our interest was to develop a general approach for CIR image segmentation that could be referred by other researchers. After preliminarily testing several approaches, the *K*-means clustering algorithm was selected. A key parameter while applying this algorithm was the selection of proper number of clusters. In this study, “3” was applied as pixels can be categorized into three clusters, namely, plant, background, and noise (Figure 5). Since three images (clusters) were randomly generated after implementing the algorithm, it was necessary to develop a solution that would automatically select the proper cluster with plants (not background or noise). In the NIR channel, plants had a stronger signal over the background and noise, which made it a good parameter to differentiate the plant cluster from noise and background clusters. The average intensity for the NIR channel of each image was calculated, and the image with the highest value was selected as the plant cluster. After small objects as noises were removed, the leaf in the CIR image was segmented, and then, the dataset was prepared for further analysis. The dataset size is shown in Table 1.

**TABLE 1:** Datasets (region of interest) of paired images and their sizes.

Leaf rust disease dataset	Number of images	Tan spot disease dataset	Number of images
Free color	226	Free color	237
Free color-infrared	226	Free color-infrared	237
Light color	171	Light color	182
Light color-infrared	171	Light color-infrared	182
Severe color	200	Severe color	200
Severe color-infrared	200	Severe color-infrared	200

### Handcrafted Features Extraction

Based on domain knowledge and reported results (Lu and Lu, 2018; Wang et al., 2019), color, vegetation fraction, and texture were the extracted HFs features in this study. Although both color and CIR images contain red and green channels, based on our preliminary comparisons of the same channel images from two cameras, they were not exactly the same (probably due to slight differences in band center and width of the two cameras).

For the color images in RGB format, they were also converted into HSI (hue, saturation, intensity) and Lab formats, and the normalized average intensity of each channel was calculated (a total of 9 HFs). For the CIR images, since the original image consists of 3 channels (e.g., R, G, and NIR), the normalized average intensity of each channel was calculated. Then, the CIR image was converted into HSI and Lab formats, with another 6 color features obtained (a total of 9 HFs).

After extracting the color features, the vegetation fraction features were extracted. For the color image, it included Normalized Difference Index (NDI), Excess Green (E × G), Excess Red (E × R), Color Index of Vegetation Extraction (CIVE), Modified Excess Green (ME × G), and Normalized Excess Green (NE × G). For the CIR image, due to the lack of blue channel information, the features of E × G, CIVE, ME × G, and NE × G could not be extracted. However, with the NIR channel information, NDVI and Green Normalized Difference Vegetation Index (GNDVI) features were obtained. For the texture features, they were extracted using the gray-level co-occurrence matrix (GLCM), including correlation, contrast, dissimilarity, energy, entropy, and homogeneity. Details of all the features extracted, as well as their calculation formulas, have been described in Wood et al. (2012); Wang et al. (2019), and Aballa et al. (2020).

### Deep Features Extraction

In contrast to HFs that require domain knowledge to decide which type of features to extract, the extraction of DFs is domain knowledge-free. Although different trained CNNs can be used to extract DFs, it was unknown which one could extract appropriate DFs that can better represent crop diseases. Hence, several DL models, including AlexNet, VGG16, ResNet101, GoogLeNet, and Xception, were used to extract DFs, which were then fed into a classifier to select the one with the highest accuracy. Since model training is time-consuming, this study took advantage of these trained models for DFs extraction (Aballa et al., 2020). Since each model has many deep layers (consisting of CNNs and fully connected layers), it was necessary to decide which layer to use for DFs extraction. Since previous studies demonstrated that shallow layers mainly reserved spatial and general shape information (might not be significantly related to disease detection) (Jiang and Li, 2020), this study extracted DFs using the layer before the last fully connected layer of each model. The layer name and the number of extracted DFs for each model are presented in Table 2.

**TABLE 2:** Information of deep learning models used for extracting deep features.

Model information	AlexNet	VGG16	ResNet101	GoogLeNet	Xception
Number of deep layers	8	16	101	22	71
Feature pooling layer name	drop7	drop7	pool5	pool5-drop_7 × 7_s1	avg_pool
Number of features extracted	4,096	4,096	2,048	1,024	2,048

### Parallel Feature Fusion

Many data fusion techniques have been used to improve model accuracy. One approach is to first register images from different sources and then fuse them using the Laplacian Pyramid Transform (LPT) or Fuzzy Logic into one composite image (Bulanon et al., 2009). Then, the extracted features from the composite images are fed into classifiers. In another approach, on a first step decisions based on different feature sets (from different types of images) are made, after which the decisions are reconciled or combined to generate a global decision (decision fusion) (Peli et al., 1999; Yang et al., 2003). In yet another approach, features from different types of images are fused parallelly (concatenated) and then fed into classifiers (Yang et al., 2003; Khan et al., 2018). In our case, preliminary tests revealed that the first method resulted in a poor performance, which was probably caused by the loss of color information during the fusion process. Since the second approach has not been extensively used and the performance is unavailable, we decided to use the third method for its robustness and proved performance, which included all the information from both types of images (Khan et al., 2018).

### Feature Selection and Classifier

The relevance of extracted features (HFs or DFs) for the classification is unknown beforehand. Feeding irrelevant features to the model would decrease the accuracy, as well as increase the computation load. To select relevant features, ReliefF algorithm was applied to calculate the weights of individual features (Kononenko et al., 1997). The ReliefF algorithm uses *K* nearest neighbors (KNN) for the weight calculation (the study used *k* = 3). The higher the weight of a feature, the more relevant it is to the classification. Since a negative weight indicates an insignificant role, this study used only features with positive weights for modeling.

A large number of classifiers have been used in addressing classification issues, including SVM, neural network (NN), random forest (RF), and KNN. Among these classifiers, an SVM classifier (multi-class) (Aballa et al., 2020; Zhang et al., 2020a) was selected because many studies have shown that it outperformed others (Sajedi et al., 2019; Jahan et al., 2020). For the diagnosis of different types of diseases (e.g., leaf rust, tan spot, and leaf rust + tan spot), the dataset was randomly partitioned into training (80%) and testing (20%) for model development.

In this study, Python language (V3.8) was used to develop the semiautomatic webtool (see text footnote 1) to assist paired image dataset creation (Figure 4). For all other data processing (e.g., image segmentation, feature extraction, and model development and execution), MATLAB^®^ 2019a (The Mathworks, Inc., Natick, MA, United States) was used. A desktop computer was used for data processing, which was configured with Windows 10 OS, Intel(R) Core(TM) i7-8700 CPU, 32 GB RAM, Intel(R) UHD Graphics 630, and 16 G GPU memory.

## Results and Discussion

### Diagnosis Accuracies Based on Handcrafted Features

**FIGURE 6:**
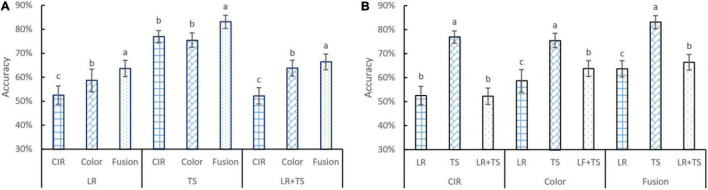
Accuracy performance of features from color and color-infrared image, and their parallel fusion on leaf rust (LR), tan spot (TS), and LR + TS (two diseases combination) detection in terms of different image types (A) and diseases (B). Whiskers on bars represent two standard deviations calculated from 20 replicates. Bars with different letters are significantly different by Tukey’s test at a significance level of 0.05.

Disease detection accuracies based on HFs from different types of images (color and CIR) and their parallel fusion are shown in Figure 6. For the statistical analysis and visualization of the whole data, the effects of features from different types of images for the same disease (Figure 6A) and the effects of disease type for the features extracted from a certain type of image (Figure 6B) are presented. For leaf rust and leaf rust + tan spot (Figure 6A) disease diagnosis, the CIR (accuracy about 53%) did not perform as satisfactorily as the color images (accuracy about 60%). One possible reason was that the leaf rust disease’s symptoms were relatively small in size, and the difference between the diseased and healthy regions was unobvious of the CIR images. This assumption is supported by the results regarding tan spot disease, where the performance of CIR and color images was not significantly different (Figure 6A), as the tan spot symptoms were large and more obvious, hence identified with a better accuracy (about 75%). Compared with the single-source features from CIR or color images, for all the three types of diseases, the parallelly fused features resulted in the highest accuracies. Since one type of image can only represent partial information, the parallel fused features from images collected by both cameras provided more meaningful information and features that best described the diseases. For the leaf rust, tan spot, and leaf rust + tan spot, the accuracy improvement using parallelly fused features over CIR image features was 21, 8, and 27%, respectively, and over color image features was 9, 10, and 4%, respectively. Such good accuracy improvements indicate the superiority of applying parallel feature fusion techniques for disease diagnosis.

Regarding detection accuracy for different diseases (Figure 6B) with the same type of features, it can be observed that tan spot was consistently the disease detected with the highest accuracy across the three types of features. The obvious difference of the tan spot symptoms from the healthy portion of the leaves and large area of discolored tissues might be the reason for the more accurate detection. On the contrary, the symptoms of leaf rust are usually small in discolored area (not significant). The overall accuracy for the leaf rust + tan spot is a little higher than that of the leaf rust alone, which is because the combined dataset contained the tan spot disease sub-dataset as well. For the CIR features, color features, and parallelly fused features, the tan spot detection accuracies were 46, 28, and 31% higher over leaf rust, respectively, and 47, 18, and 25% higher over leaf rust + tan spot, respectively.

**FIGURE 7:**
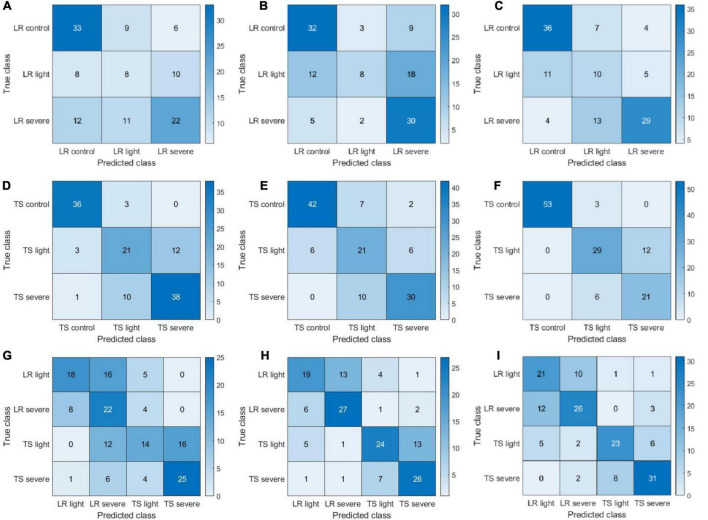
Confusion matrices of color-infrared (CIR) image features, color image features, and parallelly fused features on leaf rust (LR), tan spot (TS), and LR + TS (their combination) disease detection based on handcrafted features. (A–I) Represent a subset of three confusion matrices for CIR image features, color image features, and parallelly fused features on LR, TS, and LR + TS disease detection, respectively.

The confusion matrices presented in Figure 7 provide more detailed information on the classification/misclassification results. For the leaf rust disease severity detection, the model had difficulties in classifying the leaf rust light (light disease) correctly. A total of 31 cases (8, 12, and 11 from Figures 7A–C, respectively) of leaf rust light were misclassified as leaf rust control (disease-free), and a total of 33 cases (10, 18, and 5 from Figures 7A–C, respectively) of leaf rust light were miscategorized as leaf rust severe. The light condition may have played a big role on those results, making it very difficult to accurately assess the disease occurrence and severity. Our findings further demonstrated that disease detection on its early stages of development is challenging, which is supported by previous literature reports (Singh and Misra, 2017). For the tan spot disease detection, the major misclassifications occurred as light disease predicted as severe (30 cases; 12, 6, and 12 from Figures 7D–F, respectively). The difficulties can further support the previous assessment that it is a challenge to accurately detect and assess severity on its early stage of infection. The results (Figures 7G–I) showed a good performance in identifying the disease type (tan spot or leaf rust). For the CIR features, color features, and parallelly fused features, the disease misclassification rates (leaf rust classified as a tan spot or tan spot classified as leaf rust) were 19% (28 cases in Figure 7G), 11% (16 cases in Figure 7H), and 9% (14 cases in Figure 7I), respectively. Thus, the parallelly fused features have a more satisfactory performance in disease type identification. This piece of information is critical for researchers and farmers to select proper chemicals for disease management.

### Model Selection for Deep Features Extraction

**FIGURE 8:**
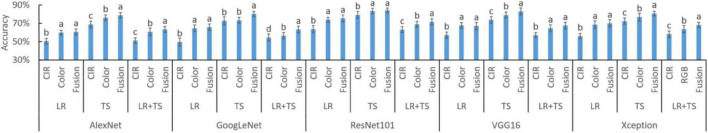
Performance of deep features (DFs) from different types of images and their parallel fusion on different wheat diseases detection. DFs were extracted by five different models. CIR, color-infrared images; LR, leaf rust; TS, tan spot; and LR + TS, combined disease datasets (without the control datasets). Whiskers on bars represent two standard deviations calculated from 20 replicates. Bars with different letters are significantly different by Tukey’s test at a 0.05 significance level.

The diagnosis accuracies based on DFs from five DL models are shown in Figure 8. For all the 15 settings (5 DL models × 3 types of diseases), the fused features resulted in the highest accuracy in eight settings (e.g., AlexNet TS and LR + TS, GoogLeNet TS and LR + TS, ResNet101 LR + TS, VGG16 TS, and Xception TS and LR + TS, where TS and LR are tan spot and leaf rust, respectively), while for the other seven settings, fused features together with color image features lead to the highest accuracies. The results indicate that, similar to the HFs, parallelly fused deep features could increase the model accuracy over deep features from a single type of image.

**FIGURE 9:**
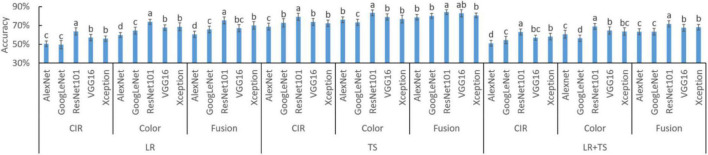
Accuracies of deep features (DFs) extracted by different deep learning models on different wheat disease diagnoses. Fusion means the parallelly fused features of color-infrared (CIR) and color images. LR, leaf rust; TS, tan spot; and LR + TS, combined disease dataset (without the control datasets). Whiskers on bars represent two standard deviations calculated from 20 replicates. Bars with different letters are significantly different by Tukey’s test at a significance level of 0.05.

To assist decision-making on which DL model should be selected for DFs extraction, the experimental results of Figure 8 were rearranged as shown in Figure 9 for better comparison of different models and statistical analysis. In each of the nine settings (3 diseases × 3 types of features), DFs extracted by ResNet101 consistently resulted in the highest accuracy (letter *a* in all 9 settings). The high diagnosis accuracy was due to the extracted features that were good representations of the crop diseases. A potential reason that the DFs by ResNet101 outperformed the DFs extracted by AlexNet, VGG16, GoogLeNet, and Xception was because the ResNet101 has more deep layers—ResNet101, AlexNet, VGG16, GoogLeNet, and Xception consist of 101, 8, 16, 22, and 71 deep layers, respectively (Table 1). With more layers, the extracted DFs could represent more detailed (fine) information of the plant diseases, while the features from shallow layers mainly reserved spatial and general information (Jiang and Li, 2020). Hence, the ResNet101 should be used for DFs extraction to serve the purpose of wheat disease diagnosis.

**FIGURE 10:**
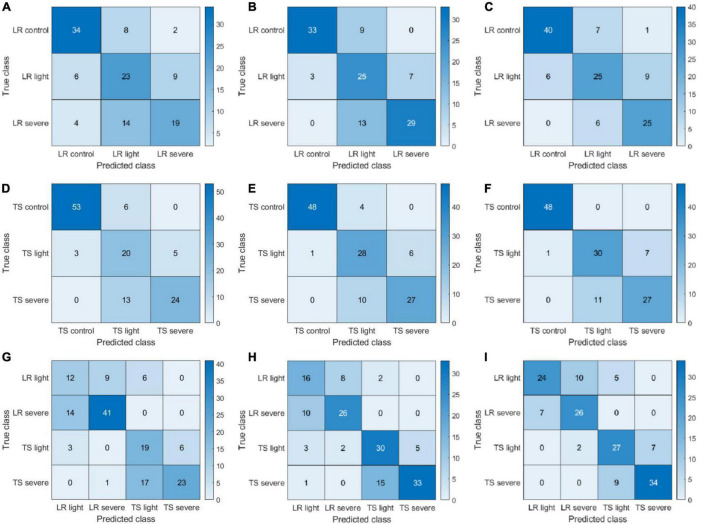
Confusion matrices of color-infrared (CIR) image deep features (DFs), color image DFs, and their parallelly fused DFs on leaf rust (LR), tan spot (TS), and LR + TS (their combination) disease detection. DFs extracted by ResNet101 and sub-figures (A–I) represent three confusion matrices of CIR HFs, color HFs, and fusion for LR, TS, and LR + TS, respectively.

Since DFs extracted by ResNet101 would lead to the highest diagnosis accuracy compared with other DL models, its performance was further studied in the form of confusion matrices to reveal the detailed classification/misclassification results (Figure 10). For the leaf rust disease diagnosis, a majority of the misclassification cases occurred as the severe infection cases predicted as light (33 cases consisting of 14, 13, and 6 cases from Figures 10A–C, respectively). The pattern of misclassification was different from the HFs as mentioned in Figure 7, where light infection cases were misclassified as severe or free of infection. The different types of misclassification indicate that the DFs represented the images differently from the HFs. For the tan spot disease diagnosis, most misclassifications that happened as severe infections were predicted as light (34 cases consisting of 13, 10, and 11 from Figures 10D–F, respectively). This type of misclassification was also different from the HFs (Figure 7), supporting the previous assessment that DFs represented the images differently from the HFs. The DF had a satisfactory performance in distinguishing the combined scenario of two diseases, and the misclassification ratios were only 6.6% (10 cases in Figure 10G), 5.3% (8 cases in Figure 10H), and 4.6% (7 cases in Figure 10I) for the CIR image DFs, color image DFs, and the parallelly fused DFs, respectively.

### Accuracy Comparison Between Handcrafted and Deep Features

**FIGURE 11:**
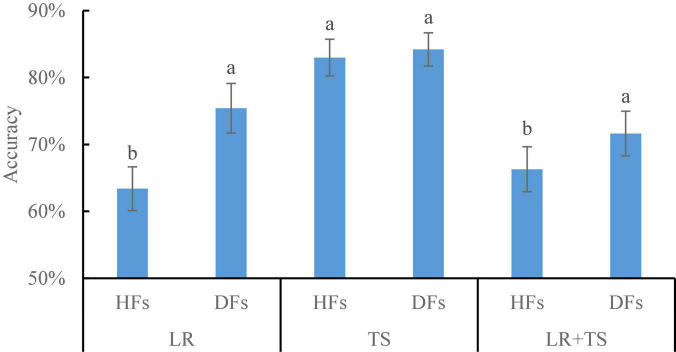
Comparison of handcrafted features (HFs) and deep features (DFs; extracted from ResNet101) on different wheat diseases detection with parallel feature fusion. Whiskers on bars represent two standard deviations calculated from 20 replicates. Bars with different letters are significantly different by Tukey’s test at a significance level of 0.05.

Results shown so far have demonstrated that parallel feature fusion could improve the model accuracy for wheat disease diagnosis for both HFs and DFs, and the DFs extracted by ResNet101 resulted in higher accuracy over the other four models, namely, AlexNet, GoogLeNet, VGG16, and Xception. To make a better assessment of that, we did a side-by-side comparison of HFs and DFs (extracted by ResNet101) on the detection of diseases using the parallelly fused features (Figure 11). For the leaf rust and leaf rust + tan spot disease diagnosis, DFs resulted in higher accuracies of 19 and 8% over HFs, respectively. A potential explanation for those results is that the symptoms for leaf rust were not very obvious (could not be manually selected), and DFs were able to extract fine features that could better represent the diseases. For the tan spot disease diagnosis, the accuracies by DFs were not significantly different from HFs, which might be because the TS symptoms were clear and obvious. Overall, it is recommended to use the DFs, instead of HFs, for wheat disease detection, coupled with the parallel feature fusion technique.

The accuracies of several wheat disease detection (e.g., smut, leaf blotch, and black chaff) studies varied greatly from 50 to 99% (Lu et al., 2017; Su et al., 2018, 2019; Wiesner-Hanks et al., 2019; Abdulridha et al., 2020; Mi et al., 2020). From this study, the recommended application of parallel fusion of CIR image DFs and color image DFs extracted from ResNet 101 resulted in the accuracies of 75, 84, and 72% for wheat leaf rust, tan spot, and leaf rust + tan spot disease detection, respectively (Figure 11). Due to the differences in the datasets applied and models employed in this study as compared with other published studies, it is nearly impossible to make a direct and objective comparison. However, the accuracy of the methodologies by Chen et al. (2018); Su et al. (2018), and Wiesner-Hanks et al. (2019) might be improved by incorporating the outcomes of this research—DFs (from ResNet101) coupled with parallel feature fusion for diagnosis accuracy improvement.

This study developed the methodology specifically for greenhouse applications. However, it also has the potential to be applied for field use in real-time mode, which can help breeders, plant scientists, and growers to obtain the wheat disease conditions. Thus, the current methodology should be tested using field data. Considering the variable lighting conditions during infield use, a color calibration/adjustment procedure at the beginning of the data process should be added to improve the model’s robustness (Sunoj et al., 2018). Furthermore, a desktop was used for the data process in this study, which should be replaced by an embedded system for infield use. Thus, a trade-off between model size, computation time, and model accuracy should be made, instead of using one parameter (accuracy) to judge the model performance.

## Conclusion

A methodology for the diagnosis of leaf rust, tan spot, and leaf rust + tan spot diseases with handcrafted and deep features from the color image, color-infrared (CIR) image, and their parallel fusion along with SVM classifier was successfully developed and compared. A webtool was developed, hosted (see text footnote 1), and used in this study for paired datasets (the same view for color and CIR images) creation. Fused features (parallel mode in this study obtained *via* concatenating) resulted in a higher disease detection accuracy over the features from a single type image (either color or CIR). It was found that deep features (automatically selected by DL algorithms with free domain knowledge) generated higher diagnosis accuracies over handcrafted features (manually selected using domain knowledge), due to extraction of fine features by DFs that would be missed by HFs. In addition, while selecting DL models for DFs extraction, it is recommended to use the efficient ResNet101 DL model generating more deep layers, as shallow features can only reserve spatial and general information. The developed methodology based on DFs and parallel feature fusion efficiently detected wheat disease conditions with accuracies of 74, 84, and 72% for leaf rust, tan spot, and leaf rust + tan spot, respectively. This methodology, which can be readily used in greenhouse applications by plant pathologists, breeders, and other users, presents a pathway toward the development of automatic and objective wheat disease diagnosis applications. Furthermore, the field application of the methodology can be achieved with further tests of field data and fine-tuning of model parameters.

This study successfully and satisfactorily segmented color and CIR images using the developed general algorithms. However, the segmentation results between color and CIR images were not compared. Future studies, such as comparing overlapping ratio, should be conducted in this field. Furthermore, the mask generated for segmentation color images should be tested on the CIR image, and vice versa. This study took advantage of ReliefF for the feature section, and under some conditions, the elimination of features may not improve the model accuracy. Therefore, future studies should compare the model accuracy between the feature selection and non-selection. This study mainly focuses on the model accuracy, and it lacks a comprehensive comparison among different models, such as training time and model size. Future research should compare the models more comprehensively. This study took advantage of the SVM as the classifier for its proven performance. Further studies should be conducted to compare the performance of different classifiers, such as neural network and random forest.

## Data Availability Statement

The raw data supporting the conclusions of this article will be made available by the authors, without undue reservation.

## Author Contributions

ZZ contributed to conceptualization, data curation, formal analysis, funding acquisition, investigation, writing original draft, supervision, reviewing, and editing. PF contributed to funding acquisition, writing, reviewing, and editing. AF, ZL, and CI contributed to resources, writing, reviewing, and editing. XH and HK contributed to writing, reviewing, and editing. NJ and SS contributed to data curation. JM contributed to software. All authors contributed to the article and approved the submitted version.

## Conflict of Interest

The authors declare that the research was conducted in the absence of any commercial or financial relationships that could be construed as a potential conflict of interest.

## Publisher’s Note

All claims expressed in this article are solely those of the authors and do not necessarily represent those of their affiliated organizations, or those of the publisher, the editors and the reviewers. Any product that may be evaluated in this article, or claim that may be made by its manufacturer, is not guaranteed or endorsed by the publisher.

## Footnotes

1
https://github.com/jithin8mathew/RGB_CIR_imageCropping_tool
