# Supplementary material for: Enhancing Wheat Disease Diagnosis in a Greenhouse Using Image Deep Features and Parallel Feature Fusion
Source: Front Plant Sci. 2022 Mar 10;13:834447. doi: 10.3389/fpls.2022.834447 (PMC8965652; doi:10.3389/fpls.2022.834447)
Supplement: Supplementary file 1 [file Data_Sheet_1.docx]

# Appendices

## Appendix I: Leaf rust disease grades

In this study, the modified Cobb Scale (below figure) was used to evaluate the wheat leaf rust disease severity.


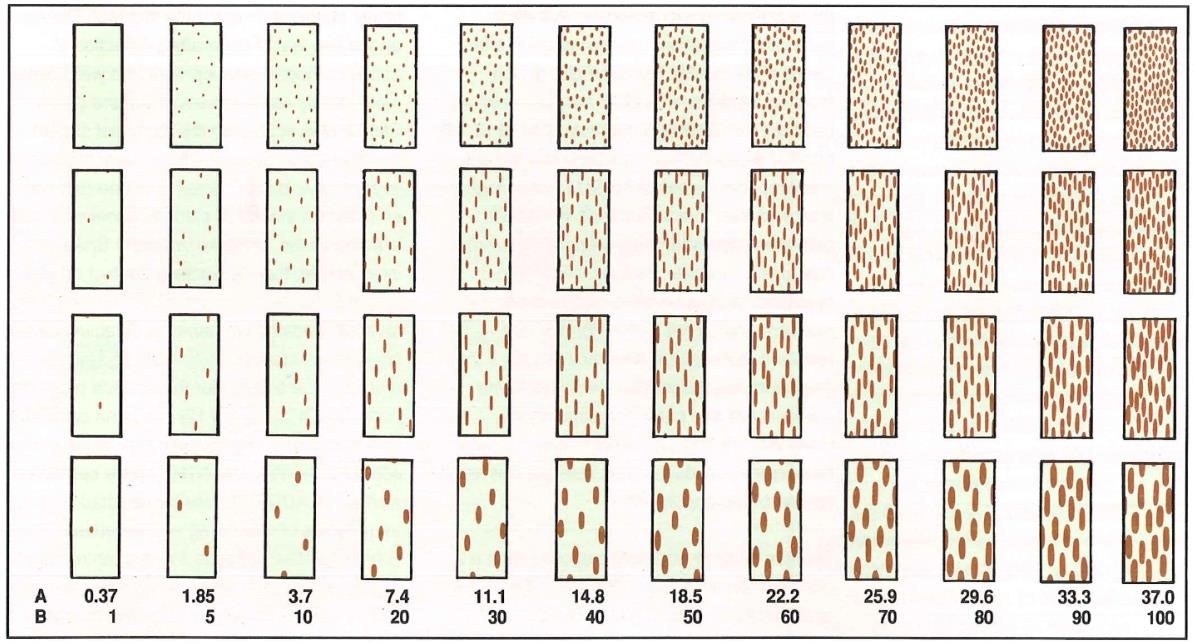


The modified Cobb Scale: (A) Actual percentage occupied by rust uredinial; (B) Rust severities of the modified Cobb Scale.

Based on the above standards, all collected images were classified into three grades: control (disease free), disease light (*modified Cobb Scale* B ≤ 10), and disease severe (*modified Cobb Scale* B *>* 10). Below figure shows examples of different leaf rust disease severity.


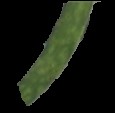

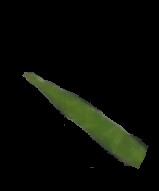

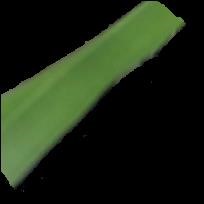

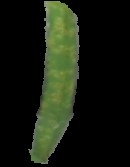

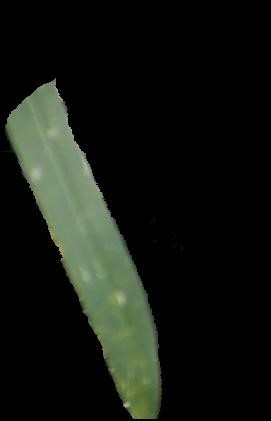

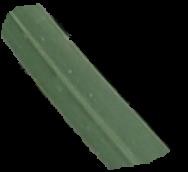


(A) (B) (C)

Examples of leaf rust disease severity grades: (A) control, (B) light, and (C) severe.

## Appendix II: Tan spot disease grades

Images collected of tan spot disease were categorized into three groups: control (disease free), disease light (discolored portion ≤ 30%), and disease severe (discolored portion *>* 30%). Examples are shown in below figure.


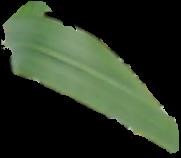

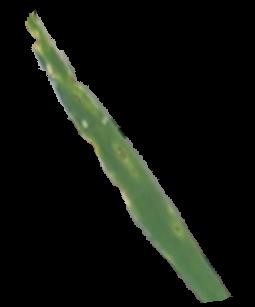

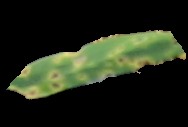

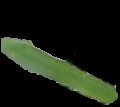

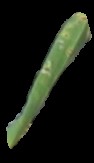

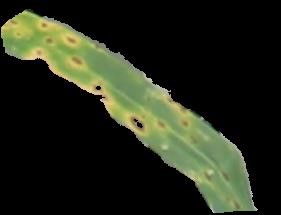


(A) (B) (C)

Examples of tan spot disease severity grades: (A) control, (B) light, and (C) severe.
